# Interaction of High-Density Polyethylene Microplastics with Freshwater Microalgae *Chlorella vulgaris* and *Scenedesmus quadricauda*

**DOI:** 10.3390/ijms27156812

**Published:** 2026-07-29

**Authors:** Alicja Faszczewska, Alicja Piotrowska-Niczyporuk, Urszula Klekotka, Joanna Karpińska

**Affiliations:** 1 Doctoral School of Exact and Natural Sciences, University of Bialystok, Ciolkowskiego 1K, 15-245 Bialystok, Poland; a.faszczewska@uwb.edu.pl; 2 Department of Plant Biology and Ecology, Faculty of Biology, University of Bialystok, Ciolkowskiego 1J Street, 15-245 Bialystok, Poland; 3 Department of Materials Chemistry, Faculty of Chemistry, University of Bialystok, Ciolkowskiego 1K Street, 15-245 Bialystok, Poland; u.klekotka@uwb.edu.pl; 4 Department of Analytical and Inorganic Chemistry, Faculty of Chemistry, University of Bialystok, Ciolkowskiego 1K Street, 15-245 Bialystok, Poland; joasia@uwb.edu.pl

**Keywords:** antioxidants, HDPE, microalgae, microplastics, oxidative stress

## Abstract

The objective of the present study was to evaluate the impact of high-density polyethylene (HDPE) used in two particle sizes (40 and 125 µm) on selected biochemical parameters in two species of green algae: *Chlorella vulgaris* and *Scenedesmus quadricauda*. Exposure to microplastics resulted in decreased cell numbers (up to 43%) and reductions in protein (up to 42%), monosaccharide, and chlorophyll contents in both algal species. Oxidative stress, as reflected by increased hydrogen peroxide levels (up to 120%) and enhanced lipid peroxidation, was observed in HDPE-treated cells. This was accompanied by elevated antioxidant enzyme activity, changes in ascorbate and proline content, and alterations in carotenoid composition. Thus, toxicity of HDPE and the induction of defense mechanisms involved in adaptation to environmental pollutants were confirmed in both microalgae. Changes in the physicochemical properties of the tested polymer surface were investigated using Fourier-Transform Infrared Spectroscopy, scanning electron microscopy (SEM), and porosimetry analysis. The presence of biofilm, hydroxyl groups, higher total pore volume, and maximum pore width on the surface of the microplastic confirmed that green algae may be responsible for the first step in HDPE structure modifications. Understanding the interactions between such micropollutants and green algae, key primary producers in aquatic food webs, is therefore essential.

## 1. Introduction

The term ‘microplastics’ (MPs) typically refers to plastic particles less than 5 mm in size. MPs have become ubiquitous and persistent contaminants in aquatic environments [1,2]. These particles arise from primary sources and from the fragmentation of larger plastic debris, leading to their widespread distribution in freshwater and marine systems [3,4,5]. High-density polyethylene (HDPE) is a frequently detected polymer, with an estimated concentration of about 2.5 mg L^−1^ in freshwater ecosystems [6,7]. HDPE particles of 40 and 125 µm are commonly used in commercial applications due to their high resistance to degradation. Consequently, HDPE persists in the environment for extended periods [7,8]. The physicochemical properties of MPs, including their small size, hydrophobicity, and large surface area, facilitate interactions with living organisms and enhance their bioavailability. Particle size is also a major determinant of the extent of MP interactions with microorganisms, as it can influence particle adhesion to cell surfaces, biofilm formation, and the intensity of physiological stress responses. As a result, different particle sizes may differ not only in their toxic effects but also in their susceptibility to biological transformation and biodegradation [3,9,10,11,12]. Consequently, MPs can induce a range of deleterious effects, including oxidative stress and metabolic disturbances, particularly in primary producers directly exposed to suspended particles [13,14,15]. In this context, increasing attention has been given to the potential role of microalgae in the removal and transformation of microplastics from aquatic systems [8,16,17,18].

Microalgae are primary producers in aquatic ecosystems. They play a fundamental role in nutrient cycling and oxygen production [19,20,21]. However, they are also directly exposed to suspended contaminants, including MPs [22]. Microalgae can interact with plastic particles through surface colonization, biofilm formation, and aggregation. These interactions may influence particle distribution in the medium and their potential removal from aquatic systems [23]. Consequently, microalgae have attracted increased research interest as bioindicators of environmental pollutants and as potential agents for the biodegradation of microplastics in contaminated waters [24,25,26,27].

Despite the growing number of studies addressing the influence of MPs on microalgal viability [28,29,30,31,32], the mechanisms underlying their effects on the physicochemical parameters of the microplastic surface remain poorly understood. In particular, there is a paucity of information on the effects of exposure to specific polymers, such as HDPE, on key biochemical parameters, including proteins, carbohydrates, photosynthetic pigments, and stress-related responses, in two green microalgae with different cell structures. Some research has focused on the effects of different MPs on algal growth, metabolite levels, oxidative stress, and antioxidant defense systems [33,34]; however, the effects of HDPE on test organisms, such as *Chlorella vulgaris* and *Scenedesmus quadricauda*, which are the two major algal species in freshwater environments and are recognized as bioindicators of water quality, have not been studied yet. *C. vulgaris* lives exclusively as single, microscopic cells, and its cell wall is smooth. *S. quadricauda*, on the other hand, forms permanent colonies, most often composed of four or eight cells arranged linearly in a single row. The outermost cells of *S. quadricauda* colonies have long, thread-like spines at their poles [23,33,34,35,36,37]. Because particle size directly affects surface characteristics and the available contact area between MPs and algal cells, it may influence both the magnitude of toxic responses and the efficiency of algal-mediated chemical modifications. However, comparative information on different HDPE particle sizes remains limited. Therefore, the growth and cellular responses of two algal species to MPs applied at two particle sizes (40 and 125 µm) may differ, and these two algae may exert different influences on the physicochemical properties of the HDPE surface.

The aim of this study was to investigate the effects of two HDPE particle sizes (40 and 125 µm) on the growth and biochemical responses of two freshwater microalgal species, *C. vulgaris* and *S. quadricauda*. The present study focused on alterations in the composition of proteins, monosaccharides, and photosynthetic pigments, as well as oxidative stress markers, enzymatic and non-enzymatic antioxidant activity. Additionally, scanning electron microscopy (SEM), Fourier-transform infrared spectroscopy (FTIR), and porosimetry measurements were utilized to characterize the potential modifications in MP structure after cultivation with algal cells.

## 2. Results

### 2.1. Algal Growth

**Figure 1:**
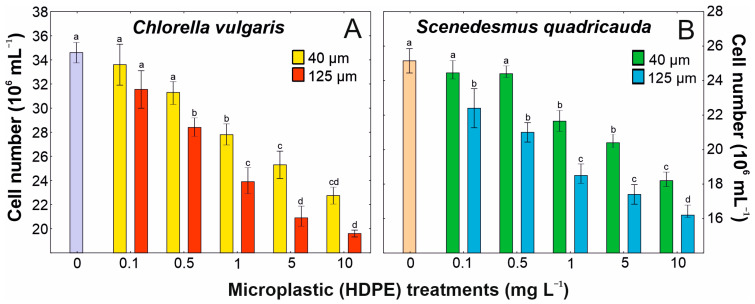
The effect of HDPE (40 and 125 µm) on the growth of *Chlorella vulgaris* (**A**) and *Scenedesmus quadricauda* (**B**) on the 5th day of cultivation relative to the control. The results show the mean values of four independent determinations, along with their respective standard deviations. Treatment with at least one letter the same is not significantly different according to Tukey’s post hoc test.

Changes in the growth of two algal species, *C. vulgaris* (Figure 1A) and *S. quadricauda* (Figure 1B), under the influence of both particle sizes (40 µm and 125 µm) of HDPE were observed on the 5th day of cultivation. HDPE at concentrations of 0.1–10 mg L^−1^ used in this work were selected to span the range from the lowest algal exposure to moderate and high HDPE doses. MP at concentrations of 0.1–0.5 mg L^−1^ did not exert a statistically significant effect on cell number, which can be treated as an algal growth parameter. HDPE at concentrations of 1, 5, and 10 mg L^−1^ reduced the growth of both algal species, with the toxic effect proportional to the microplastic concentration. For example, 40 µm HDPE and 125 µm HDPE at the highest concentration of 10 mg L^−1^ reduced the cell number of *C. vulgaris* by 34% and 43%, whereas 40 µm HDPE and 125 µm HDPE at 10 mg L^−1^ inhibited *S. quadricauda* growth by 28% and 37%, respectively, in comparison with the control culture.

### 2.2. Protein and Monosaccharide Content

**Figure 2:**
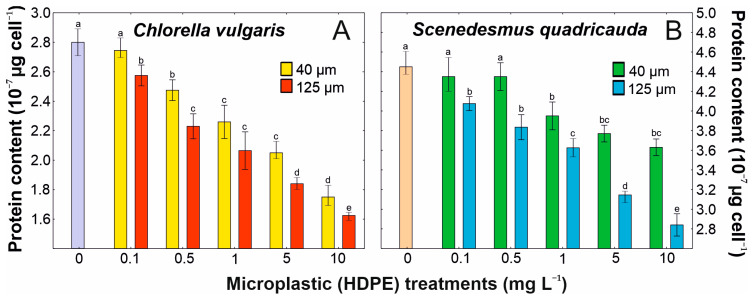
The effect of HDPE (40 and 125 µm) on protein content in *Chlorella vulgaris* (**A**) and *Scenedesmus quadricauda* (**B**) cells on the 5th day of cultivation relative to the control. The results show the mean values of four independent determinations, along with their respective standard deviations. Treatment with at least one letter the same is not significantly different according to Tukey’s post hoc test.

**Figure 3:**
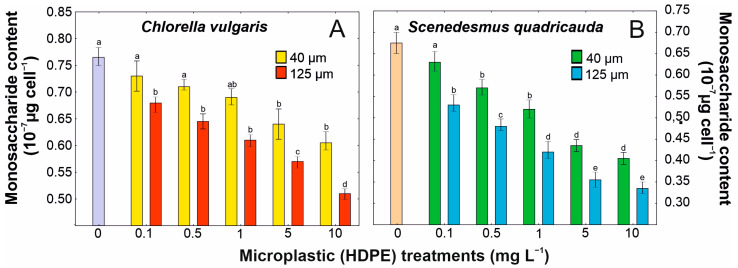
The effect of HDPE (40 and 125 µm) on monosaccharide content in *Chlorella vulgaris* (**A**) and *Scenedesmus quadricauda* (**B**) cells on the 5th day of cultivation relative to the control. The results show the mean values of four independent determinations, along with their respective standard deviations. Treatment with at least one letter the same is not significantly different according to Tukey’s post hoc test.

The presence of MPs greatly influences the accumulation of biochemical constituents, such as total soluble proteins and monosaccharides, in both algal species on the 5th day of the experiment. Exogenous application of both 40 µm HDPE and 125 µm HDPE particles at the highest concentration, 10 mg L^−1^, decreased soluble protein content by 37% and 42% in *C. vulgaris* cells compared to the control (Figure 2A), whereas the decrease in protein content was 19% and 36% in *S. quadricauda* cultures (Figure 2B) exposed to smaller (40 µm) and larger (125 µm) HDPE particle sizes applied at the highest concentration, 10 mg L^−1^, respectively. The monosaccharide level decreased by 21% and 33% in *C. vulgaris* cells in response to smaller (40 µm) and larger (125 µm) HDPE particles, respectively, applied at the highest concentration of 10 mg L^−1^, relative to control cultures (Figure 3A). Similarly, *S. quadricauda* cultures (Figure 3B) were characterized by lower sugar contents by 40–50% in response to both HDPE particles used at the highest concentration of 10 mg L^−1^ in comparison with the control.

### 2.3. Photosynthetic Pigment Content

**Figure 4:**
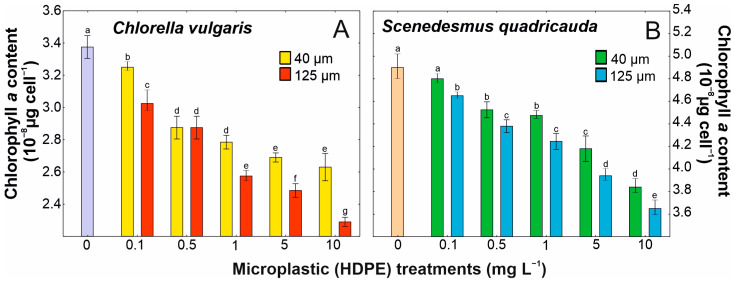
The effect of HDPE (40 and 125 µm) on chlorophyll *a* content in *Chlorella vulgaris* (**A**) and *Scenedesmus quadricauda* (**B**) cells on the 5th day of cultivation relative to the control. The results show the mean values of four independent determinations, along with their respective standard deviations. Treatment with at least one letter the same is not significantly different according to Tukey’s post hoc test.

**Figure 5:**
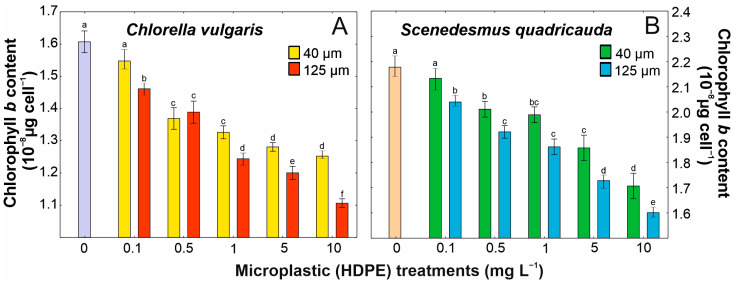
The effect of HDPE (40 and 125 µm) on chlorophyll *b* content in *Chlorella vulgaris* (**A**) and *Scenedesmus quadricauda* (**B**) cells on the 5th day of cultivation relative to the control. The results show the mean values of four independent determinations, along with their respective standard deviations. Treatment with at least one letter the same is not significantly different according to Tukey’s post hoc test.

Changes in photosynthetic pigment levels were examined in *C. vulgaris* and *S. quadricauda* cultures exposed to HDPE particles (40 and 125 µm) on the 5th day of cultivation. A decrease of 22–32% in chlorophyll *a* (Figure 4A) and of 21–27% in chlorophyll *b* (Figure 5A) was observed in *C. vulgaris* cultures treated with 40 µm and 125 µm HDPE at the highest selected concentration of 10 mg L^−1^. Moreover, a similar toxic effect of HDPE on the photosynthetic apparatus was observed in *S. quadricauda* cells, with inhibition of chlorophyll *a* (Figure 4B) by 22–26% and of chlorophyll *b* (Figure 5B) by 22–27% in response to 10 mg L^−1^ 40 µm and 125 µm HDPE, respectively.

On the other hand, exposure to 10 mg L^−1^ HDPE, particularly to smaller HDPE particles (40 µm), increased the content of *α*- and *β*-carotene, carotenes, and xanthophylls such as astaxanthin, cryptoxanthin, lutein, neoxanthin, violaxanthin, and zeaxanthin in both algal species, *C. vulgaris* and *S. quadricauda*, on the 5th day of the experiment (Table 1).

### 2.4. Oxidative Stress

**Figure 6:**
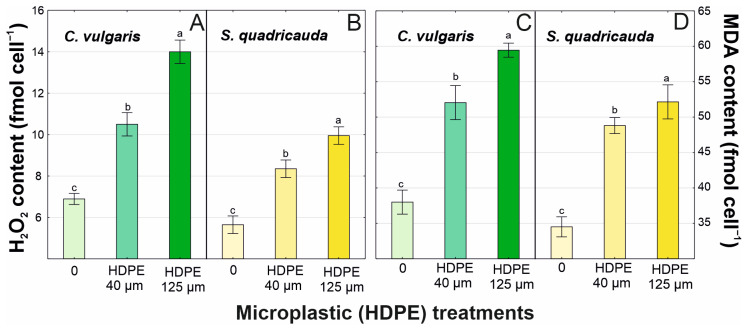
The effect of 10 mg L^−1^ HDPE (40 and 125 µm) on hydrogen peroxide (H_2_O_2_) level (**A**,**B**) and malondialdehyde (MDA) (**C**,**D**) level in *Chlorella vulgaris* and *Scenedesmus quadricauda* on the 5th day of cultivation relative to control. The results show the mean values of four independent determinations, along with their respective standard deviations. Treatment with at least one letter the same is not significantly different according to Tukey’s post hoc test.

HDPE at the highest concentration 10 mg L^−1^ induced oxidative stress in both algal species *C. vulgaris* and *S. quadricauda*, leading to a high amount of H_2_O_2_ production and lipid peroxidation expressed as malondialdehyde (MDA) level on the 5th day of cultivation (Figure 6).

Increases of 76% and 120% in H_2_O_2_ levels were observed in *C. vulgaris* (Figure 6A) exposed to 40 µm and 125 µm HDPE, respectively, compared with the control. *S. quadricauda* showed higher H_2_O_2_ levels by 46% and 75% in response to 40 µm and 125 µm HDPE, respectively (Figure 6B). MDA content, a specific marker of cell membrane damage caused by lipid peroxides, increased significantly in algal cells in the presence of both HDPE particle sizes. Thus, MDA levels were stimulated by 37% and 56% in *C. vulgaris* (Figure 6C) treated with 40 µm and 125 µm HDPE, and by 41% and 51% in *S. quadricauda* (Figure 6D) grown in the presence of 40 µm and 125 µm HDPE, respectively, relative to controls.

### 2.5. Ascorbate and Proline Levels

**Figure 7:**
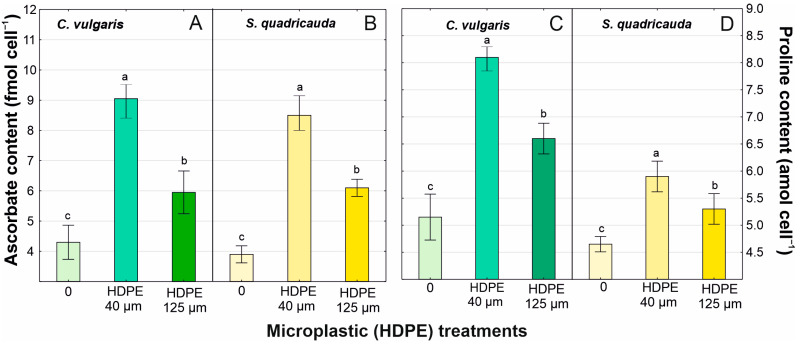
The effect of HDPE (40 and 125 µm) on ascorbate (**A**,**B**) and proline (**C**,**D**) levels in *Chlorella vulgaris* and *Scenedesmus quadricauda* cells on the 5th day of cultivation relative to the control. The results show the mean values of four independent determinations, along with their respective standard deviations. Treatment with at least one letter the same is not significantly different according to Tukey’s post hoc test.

Ascorbate levels increased in *C. vulgaris* cells (Figure 7A) exposed to both particle sizes, 40 µm and 125 µm HDPE applied at the highest concentration of 10 mg L^−1^, by 110% and 37%, respectively, on the 5th day of the experiment. *S. quadricauda* (Figure 7B) were also characterized by higher contents of this antioxidant by 118% and 56% in response to 40 µm and 125 µm HDPE, respectively, in comparison with the control on the 5th day of cultivation. The presence of HDPE in mineral medium significantly increased the ascorbic acid production in both algal species in the current study. Additionally, proline levels were stimulated by 27% and 58% in *C. vulgaris* (Figure 7C), and by 27% and 14% in *S. quadricauda* (Figure 7D) under the influence of 40 µm and 125 µm 10 mg L^−1^ HDPE, respectively.

### 2.6. Antioxidant Enzyme Activities

**Figure 8:**
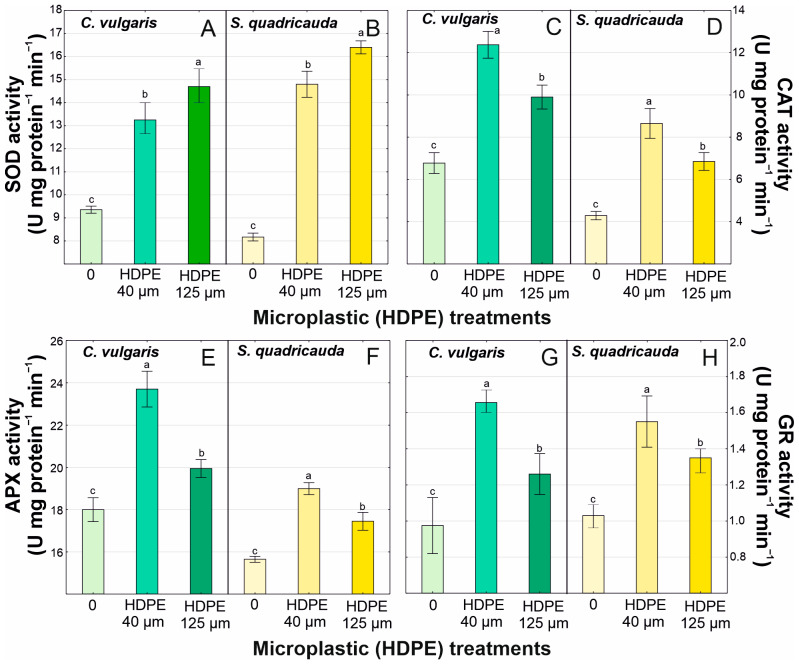
The effect of HDPE (40 and 125 µm) on SOD (**A**,**B**), CAT (**C**,**D**), APX (**E**,**F**), and GR (**G**,**H**) activity in *Chlorella vulgaris* and *Scenedesmus quadricauda* cells on the 5th day of cultivation relative to the control. The results show the mean values of four independent determinations, along with their respective standard deviations. Treatment with at least one letter the same is not significantly different according to Tukey’s post hoc test.

The induction of oxidative stress expressed as higher H_2_O_2_ and MDA levels in algal cells in response to HDPE is associated with the enhanced activity of antioxidant enzymes, including superoxide dismutase (SOD) (Figure 8A,B), catalase (CAT) (Figure 8C,D), ascorbate peroxidase (APX) (Figure 8E,F), and glutathione reductase (GR) (Figure 8G,H). This statement is confirmed by our results, showing that the presence of smaller particle size of 40 µm HDPE in the medium increased the activity of SOD by 41% and 81%, CAT by 83% and 102%, APX by 31% and 21%, and GR by 11% and 50%, in *C. vulgaris* and *S. quadricauda*, respectively, compared with the control culture on the 5th day of cultivation. On the other hand, a larger size (125 µm) of HDPE particle had a different effect on antioxidant enzyme activities in relation to 40 µm HDPE, because SOD activity was increased by 57% and 101%, CAT by 46% and 60%, APX by 11% and 12%, and GR by 29% and 31% in in *C. vulgaris* and *S. quadricauda*, respectively, in comparison with control cultures on the 5th day of cultivation.

### 2.7. Physicochemical Characterization of HDPE

**Figure 9:**
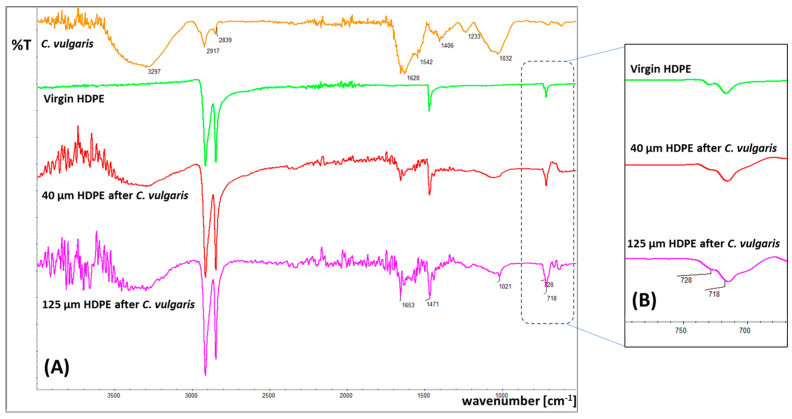
FTIR spectra of (**A**) *C. vulgaris*, virgin HDPE, 40 µm HDPE and 125 µm HDPE after cultivation with *C. vulgaris* on the 5th day of the experiment, (**B**) zoom on 720–730 cm^−1^ range.

**Figure 10:**
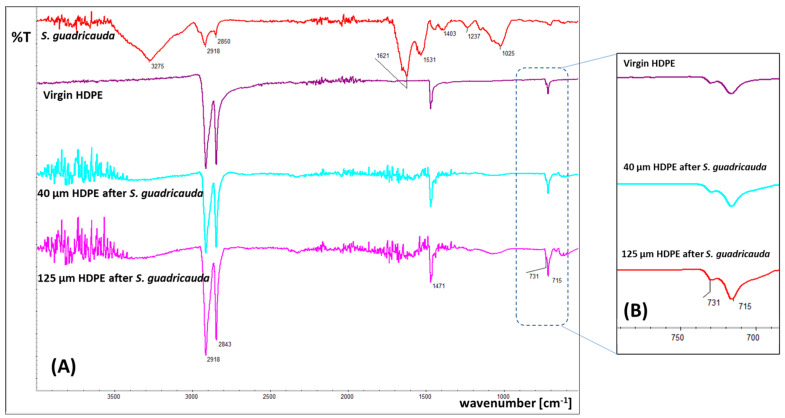
FTIR spectra of (**A**) *S. quadricauda*, virgin HDPE, 40 µm HDPE and 125 µm HDPE after cultivation with *S. quadricauda* on the 5th day of the experiment, (**B**) zoom on 720–730 cm^−1^ range.

Figure 9 and Figure 10 present FTIR spectra of dried *C. vulgaris* and *S. quadricauda,* virgin HDPE, and 40 µm and 125 µm HDPE after cultivation with *C. vulgaris* and *S. quadricauda* on the 5th day of the experiment. Prior to the measurement, the HDPE was washed 3 times with distilled water to remove the biofilm that had formed. Therefore, the presented spectra show only the structure of the HDPE surface. FTIR spectra of *C. vulgaris* (Figure 9) and *S. quadricauda* (Figure 10) presents macromolecular blueprint of the biomass, where bands typical for -OH (3300 cm^−1^), symmetric and asymmetric vibrations of -C-H (2917–2839 cm^−1^), C=O and N-H from peptide bands (1630 and 1540 cm^−1^), C-H groups from proteins (1406 cm^−1^), P=O bonds from nucleic acids (1240 cm^−1^), and C-O from cellulose (1030 cm^−1^) are observed. FTIR of virgin HDPE shows signals typical for C-H (2917, 2847, and 1471 cm^−1^), and C-C (714 and 729 cm^−1^) bonds. Comparison of reference virgin HDPE with the one after cultivation with *C. vulgaris* shows small additional signals at 1630 cm^−1^, which can be attributed either to residual biofilm on the surface of the microplastic, to the presence of a C=C stretching bond, or to vibrations of residual water bonds. A small, wide band at 1030 cm^−1^ is most likely associated with remaining biofilm on the microplastic surface. Zooming on the wavenumber range 730–720 cm^−1^ also shows an interesting effect. From the signals at 720 and 730 cm^−1^, the crystallinity index (PI) can often be calculated. In our case, the band at 730 cm^−1^ is almost absent; therefore, PI was not calculated. The disappearance of this band suggests that HDPE responded differently after exposure to *C. vulgaris* than to *S. quadricauda*, for which PI could still be determined. In the case of *S. quadricauda*, no additional signals are observed, and zooming into the range 730–720 cm^−1^ allows determination of PI indices. For virgin HDPE, the value is 0.15, while for both samples after biological aging, the value is 0.1, suggesting a decrease in crystallinity of HDPE and therefore its degradation.

**Figure 11:**
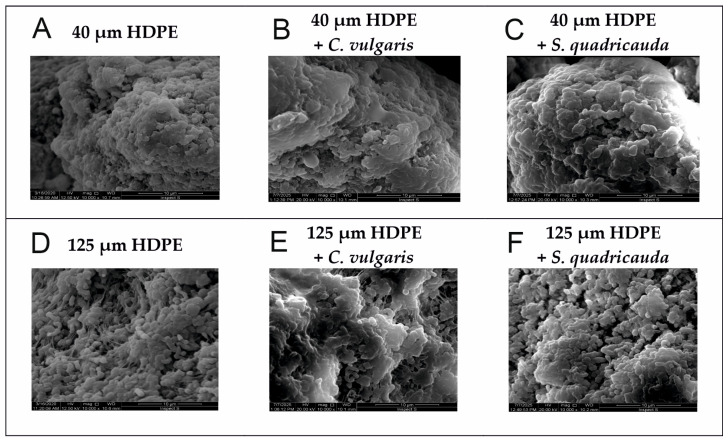
SEM images of virgin 40 µm (**A**) and 125 µm (**D**) HDPE, 40 µm and 125 µm HDPE after cultivation with *C. vulgaris* (**B**,**E**), and 40 µm and 125 µm HDPE after cultivation with *S. quadricauda* (**C**,**F**) on the 5th day of the experiment.

Comparison of the SEM images of virgin HDPE after exposure to algae (*C. vulgaris* and *S. quadricauda*) with reference HDPE shows that the MP cultured with green algae appears smoother (Figure 11), consistent with the literature suggesting biofilm formation as well as slow biological aging. The surface of HDPE contains amorphous C-C bonds, which are primarily consumed by both algal species.

Characterization of the surface properties of HDPE before and after cultivation with algae was also performed using porosity measurements and calculation of the BET (Brunauer–Emmett–Teller) specific surface area (Table 2). HDPE with a particle size of 40 µm had the lowest BET surface area (0.31 m^2^ g^−1^) and the smallest maximum pore width. After contact with *C. vulgaris*, the BET surface area of 40 µm HDPE particles increased significantly (to 5.97 m^2^ g^−1^), suggesting that this algal species had altered the MP surface. For HDPE with a particle size of 125 µm, the BET surface area was 3.54 m^2^ g^−1^ and did not change significantly after contact with the corresponding algae. After contact with *S. quadricauda*, a slight increase in BET surface area was observed, but this effect was not significant. More pronounced changes were observed in the maximum pore width parameter. In all cases, this value more than doubled, further indicating changes in the microplastics’ surface morphology.

## 3. Discussion

The green microalgae *C. vulgaris* and *S. quadricauda* could serve as bioindicators of freshwater pollution, as both species exhibited distinct biochemical responses to HDPE exposure. Our results indicate that *C. vulgaris* is more sensitive to HDPE treatment than *S. quadricauda*, which may be related to its smaller cell size, higher surface-to-volume ratio, unicellular organization and differences in cell-wall structure [23,28,32,35,37]. In contrast, the colonial growth form and more robust cell wall of *S. quadricauda* may reduce direct contact with MPs and contribute to greater tolerance. Moreover, *C. vulgaris* interacted more effectively with the HDPE surface, increasing its porosity and potentially its specific surface area, which may enhance the absorption of contaminants such as heavy metals, pathogens, and organic pollutants [3,16,17,20].

The results confirmed that growth inhibition in both microalgal species was concentration-dependent. Similar reductions in algal growth after exposure to HDPE, PVC, and PS microplastics have also been reported in previous studies. Kapelewska et al. [34] reported that HDPE significantly reduced the cell number of the green alga *Acutodesmus obliquus*, with the strongest effect observed at 500 mg L^−1^. Additionally, decreases in cell number in response to 10 mg L^−1^ polyvinyl chloride (PVC) microplastics were observed in the marine diatom *Phaeodactylum tricornutum* [38] and the green alga *C. vulgaris* [39]. Polystyrene (PS) microplastic reduced *C. vulgaris* growth and colony formation, confirming its phytotoxic effect on algal cultures [40]. Zhao et al. [41] demonstrated that MPs may reduce light penetration, inhibit photosynthesis, limit nutrient availability, and negatively affect algal growth. In addition, the adhesion of plastic particles to algal cells may impair gas exchange and nutrient uptake. These mechanisms may explain the stronger inhibitory effect of 125 µm HDPE observed in the present study.

The presence of MPs reduced the accumulation of biochemical constituents, including total soluble proteins and monosaccharides, in *C. vulgaris* and *S. quadricauda* cells. Our results are consistent with previous observations showing decreases in protein content of 11% [42] and 18% [34] in *A. obliquus* exposed to 250 and 500 mg L^−1^ of HDPE, respectively. Similarly, total soluble protein content markedly decreased in *C. vulgaris* following exposure to PET microplastics [40], whereas aminopolystyrene induced protein degradation accompanied by DNA damage [43]. The reduction in protein and sugar content observed in the present study may be associated with ROS-induced protein damage and/or lower photosynthetic efficiency in both algal species [31]. In contrast, Bour et al. [44] reported no changes in protein and sugar contents in sediment-dwelling bivalves *Ennucula tenuis* and *Abra nitida*, indicating that these responses are species-dependent.

Changes in chlorophyll and carotenoid contents are highly sensitive bioindicators of algal vitality. Chlorophyll levels were reduced in *C. vulgaris* and *S. quadricauda* cultures exposed to both HDPE particles (40 and 125 µm). Similar chlorophyll degradation has been reported after exposure to PS microplastics in *C. vulgaris* [40], aminopolystyrene in *Navicula* sp. [45], and HDPE in *A. obliquus* [34]. Likewise, Wu et al. [46] demonstrated that MPs inhibited photosynthetic activity in *Chlorella pyrenoidosa* and *Microcystis flos-aquae*. The reduced chlorophyll content may result from ROS-induced peroxidation of chloroplast membranes [31,47]. This is consistent with the findings of Khoshnamvand et al. [43], who associated aminopolystyrene toxicity in *C. vulgaris* with decreased chlorophyll content, reduced photosynthetic activity, and increased ROS accumulation. In contrast, HDPE, particularly the 40 µm particles, increased the content of *α*- and *β*-carotene and xanthophylls in both algal species. As efficient antioxidants, carotenoids (carotenes and xanthophylls) protect the photosynthetic apparatus and neutralize excess ROS [42,47,48]. Therefore, carotenoid accumulation may be considered an algal defense and acclimation strategy against MP pollution in aquatic environments.

Most studies indicate that oxidative stress is altered by the presence of various MPs [31,46,49]. Our results confirmed that HDPE at the highest concentration (10 mg L^−1^) induced oxidative stress in both algal species, *C. vulgaris* and *S. quadricauda*, leading to the production of H_2_O_2_ and MDA. MPs may probably induce oxidative stress in microalgae by physically disrupting cell walls and organelles, causing localized abrasion, and blocking light. This disrupts photosynthesis, leading to electron leakage and ROS generation [50,51,52]. As ROS can damage cellular components, reduced growth rates of *C. vulgaris* and *S. quadricauda* cultures have been observed following exposure to both HDPE particle sizes. Correspondingly, the concentration of ROS in the green microalga *A. obliquus* was up to 1.5 times higher in response to HDPE at 500 mg L^−1^ than in the control [34]. PVC microplastics also triggered oxidative stress in *C. vulgaris*, increasing ROS levels and altering the activities of antioxidant enzymes [39]. Liu et al. [50] also reported a 1.8-fold increase in ROS production in *A. obliquus* cells growing in the presence of 50 mg L^−1^ PS microplastic. Enhanced lipid peroxidation was noted in *Chlamydomonas reinhardtii* exposed to aged and new PVC particles at 10 mg L^−1^ [51]. Moreover, oxidative stress disrupted cellular morphology and metabolism in *Haematococcus pluvialis* during prolonged exposure to PS microplastics [52]. The research results presented here provide further evidence of MP toxicity, which can generate oxidative stress, as evidenced by a striking increase in MDA and H_2_O_2_ levels in *C. vulgaris* and *S. quadricauda* exposed to HDPE.

Ascorbate and proline are important non-enzymatic antioxidants involved in ROS scavenging during oxidative stress, with proline additionally stabilising proteins under stress conditions [53]. Their levels increased in *C. vulgaris* and *S. quadricauda* cultures exposed to both 40 and 125 µm HDPE. Similar increases in ascorbic acid have been reported in other plants exposed to MPs, including *Capsicum annuum*, where it protected roots against oxidative damage caused by larger MP particles [54], and maize (*Zea mays*), in which activation of the ascorbate–glutathione cycle contributed to stress tolerance [55]. Likewise, higher concentrations of polypropylene (PP) microplastics increased proline content and antioxidant enzyme activities in wheat (*Triticum aestivum* L.), reducing oxidative stress [53]. Our results suggest that HDPE, particularly smaller particles, activates non-enzymatic antioxidant defenses to maintain cellular redox balance in both algal species.

The oxidative stress induced by HDPE is associated with the activation of enzymatic antioxidant systems, including SOD, CAT, APX, and GR, which can convert ROS and their metabolic products into less harmful molecules. SOD and CAT serve as the first line of defense against oxidative stress, whereas APX and GR constitute the second step of this process [53,55,56]. Antioxidant enzymatic responses provide critical insights into cellular detoxification mechanisms and are often associated with increased tolerance to environmental stresses. Previous studies have shown that PET, polyamide (PA), polymethyl methacrylate (PMMA), PVC, and PS microplastics increased antioxidant enzymes activities in different microalgal species [57,58]. Similarly, HDPE increased SOD, CAT, APX and GR activities by 19%, 32%, 27%, and 71%, respectively, in *A. obliquus* compared with the control on the 5th day of cultivation [34], while higher antioxidant enzyme activities were also reported in *Chlorella sorokiniana* exposed to PS microplastics [59]. Exposure to micro- and nanoplastics at 5 mg L^−1^ induced SOD activity in the marine microalga *Emiliania huxleyi* [60], whereas PET, polyamide 66 (PA66), and polyethylene (PE) microplastic increased SOD activity and glutathione accumulation in the coral *Acropora* sp. [61]. Likewise, PS microplastics enhanced the activities of SOD, APX, and CAT in the aquatic plant *Salvinia cucullata* [62], while Li et al. [31] demonstrated increased APX in *S. quadricauda* exposed to PS microplastics. Comparable antioxidant responses were also reported in three marine microalgae (*Chlorella marina*, *Nannochloropsis oculata*, and *Phaeodactylum tricornutum*) and two freshwater species (*C. vulgaris* and *Tetradesmus obliquus*) exposed to PS microplastics [63]. In agreement with these findings, GR activity increased in both algal species exposed to 40 and 125 µm HDPE in the present study. As a key enzyme of the ascorbate-glutathione cycle, GR regenerates reduced glutathione and contributes to cellular adaptation to MP-induced oxidative stress [64].

Present findings showed that HDPE stimulated the activity of the antioxidant enzymes and increased the levels of small-molecular-weight antioxidants (carotenoids, proline, and ascorbate), thereby enhancing ROS scavenging and improving algal tolerance to MP-induced oxidative stress. Moreover, 40 µm HDPE particles stimulated antioxidant defense mechanisms more effectively than 125 µm particles, probably because their higher surface-to-volume ratio and greater cellular bioavailability promoted stronger oxidative signaling and activation of antioxidant responses [54].

Our studies confirmed the physicochemical modifications of the HDPE surface by both algal species; however, *C. vulgaris* is more effective in this process. Interestingly, in HDPE exposed to *C. vulgaris*, the band at approximately 730 cm^−1^ became almost undetectable, preventing reliable calculation of the crystallinity index. This finding indicates that the FTIR response of HDPE differed between the two algal species and should be interpreted together with the SEM observations of biofilm formation and the remaining physicochemical analyses when assessing the initial changes occurring on the polymer surface. Similar FTIR-based evidence of microplastic surface modifications has been reported for PS exposed to six marine microalgal strains (*Picochlorum maculatum*, *Dunaliella salina*, *Amphora* sp., *Navicula* sp., *Synechocystis* sp., and *Limnospira*) [24]. Likewise, *D. salina* induced physicochemical modifications of HDPE confirmed by FTIR (a decrease in the intensity of CH/CH_2_ bands, the appearance of C=O, -OH, and C=C groups), SEM (pitting, surface degradation, and brittleness), and X-ray diffraction (a decrease in crystallinity) [65]. In *A. obliquus*, HDPE exposure increased the carbonyl index (CI) by 16% and the double bond index (DBI) by 83%; the SEM showed minor changes in the surface area of the MPs; elemental analysis indicated higher oxygen content; and the BET-specific surface area increased from 49 to 331 m^2^ g^−1^, confirming substantial surface modifications [34]. Similarly, FTIR analyses demonstrated that PE, PS, and PP microplastics altered the cell surface and biomolecular composition of *S. quadricauda* [66].

Comparison of SEM images of HDPE after exposure to algae with reference HDPE showed a smoother MP surface, consistent with previous reports describing biofilm formation. Biofilms are complex communities of microorganisms embedded in an extracellular polymeric substance matrix that promotes cell adhesion to microplastic surfaces [30,66]. Moreover, algal cell walls, rich in structural polysaccharides such as cellulose, facilitate adhesion and successful colonization of MPs [67]. These interactions are accompanied by physicochemical changes in the polymer surface, including increased hydrophilicity and oxidative modifications leading to the formation of new functional groups, as confirmed by FTIR analysis. Consequently, algal biofilms may provide a favorable environment for the colonization of other microorganisms, representing an important initial step in polymer surface modification and subsequent decomposition processes [34,67].

Characterization of morphological properties of HDPE surface before and after cultivation with *C. vulgaris* and *S. quadricauda* was also performed by porosity measurements and by calculation of BET specific surface area value. BET values showed a clear difference on the HDPE surface, which may originate from changes in the structure of this MP after interaction with algae. A significant increase in BET-specific surface area confirms microalgal modification of MP surface. Additionally, *C. vulgaris* is more effective at this process than *S. quadricauda*. The rapid growth of algal biofilm may accelerate erosion and aging of MPs, generating more pores on their surfaces and initiating the first step of HDPE biodegradation. Recent studies have indicated that *C. vulgaris* and other green algal species can remove MPs from aquatic environments [68].

## 4. Materials and Methods

### 4.1. Microalgal Strains and Cultivation Conditions

The freshwater microalgae *Chlorella vulgaris* and *Scenedesmus quadricauda* were used in this study. The strains were purchased from the SAG Culture of Algae Collection (Göttingen, Germany). Algal cultures were maintained in sterile Bold Basal Medium (BBM) adjusted to pH 7.0. The experiments were conducted under controlled conditions in a phytotron at 22 ± 1 °C with a 12:12 h light photoperiod. Illumination was provided at a photosynthetic photon flux density of 50 μmol m^−1^ s^−1^ photosynthetically active radiation (PAR), while the relative air humidity was maintained at 45 ± 5%. Microalgae were cultivated in sterilized 250 mL Erlenmeyer flasks containing 100 mL of BBM. The cultures were continuously mixed throughout the experimental period to ensure homogeneous distribution of cells and MP particles. The cultivation period lasted for 5 days, when the growth rate of algal cultures had reached a stagnation phase, and the cell number had reached its maximum value.

### 4.2. Experimental Design and HDPE Exposure

High-density polyethylene (HDPE) microplastics (Sigma-Aldrich, St. Louis, MO, USA) with particle sizes of 40 and 125 μm were used in the experiment. HDPE was added directly to algal cultures at final concentrations of 0.1, 0.5, 1, 5, and 10 mg L^−1^. For each HDPE particle size, a separate control culture without MP addition was maintained under identical cultivation conditions. Thus, each experimental series consisted of six treatment groups: control, 0.1, 0.5, 1, 5, and 10 mg L^−1^ HDPE. The experimental design was applied independently to both *C. vulgaris* and *S. quadricauda*. All treatments were prepared four times and cultivated for 5 days under the conditions described in Section 4.1. Samples were collected on the 5th day of cultivation for further analyses.

### 4.3. Determination of Algal Growth and Cell Density

Algal growth was evaluated by estimating cell density using a combination of direct microscopic counts and spectrophotometric measurements. At the beginning of the experiment (day 0), aliquots of control cultures of *C. vulgaris* and *S. quadricauda* were collected, and cell numbers were determined using a Bürker counting chamber (Witeg Labortechnik GmbH: Wertheim, Germany) under a light microscope. Simultaneously, four spectrophotometric measurements of each culture were performed at a wavelength of 680 nm. For each microalgal species, the relationship between absorbance and cell number was established based on the initial measurements. On the 5th day of cultivation, spectrophotometric measurements and direct counting of cells in Bürker counting chamber were repeated four times for all experimental variants. Cell densities were subsequently estimated using species-specific calibration relationships between absorbance and the corresponding number of cells determined on day 0 and 5th day of cultivation.

### 4.4. Determination of Soluble Proteins

The soluble protein content in algal cells was determined spectrophotometrically according to the method of Bradford [69], based on the binding of Coomassie Brilliant Blue G-250 dye to proteins. Briefly, 10 mL of thoroughly mixed algal suspension was centrifuged (3500 rpm, 10 min), and the resulting pellet was used for protein extraction. Protein determination was performed using the micromethod, in which 200 μL of diluted Bradford reagent (1:4, *v*/*v*) was added to the algal pellet. Following incubation, absorbance was measured at 595 nm against an appropriate blank. Bovine serum albumin (BSA) was used as a standard for protein quantification. Protein content was expressed per cell.

### 4.5. Determination of Reducing Sugars

The content of reducing sugars in algal cells was determined spectrophotometrically using the Somogyi–Nelson method [70]. Briefly, 10 mL of thoroughly mixed algal suspension was centrifuged (2000 rpm, 10 min), and the supernatant was discarded. The resulting pellet was extracted with 2.5 mL of ethanol and incubated at 60 °C for 24 h in the absence of light. Subsequently, samples were centrifuged again (2000 rpm, 10 min), and 0.5 mL of the obtained extract was used for further analyses. For the determination of reducing sugars, 0.5 mL of Somogyi copper reagent was added to 0.5 mL of the sample extract. The reaction mixtures were heated in a boiling water bath (100 °C) for 20 min and then cooled under running water. Afterwards, 0.5 mL of Nelson arsenomolybdate reagent and 3.5 mL of distilled water were added. The absorbance was measured at 540 nm against an appropriate blank. Glucose was used as a standard, and the reducing sugar content was expressed per cell.

### 4.6. Determination of Photosynthetic Pigments

The composition of photosynthetic pigments of *C. vulgaris* and *S. quadricauda* was determined using high-performance liquid chromatography (HPLC) [71]. Algal samples were centrifuged, and the supernatant was discarded. The resulting pellet was extracted with 1 mL of HPLC-grade methanol and homogenized using a bead mill (TissueLyser LT, Qiagen GmbH, Düsseldorf, Germany). The extracts were stored overnight at 4 °C in darkness and subsequently centrifuged. The obtained supernatants were filtered through 0.45 μm PTFE syringe filters (A&A Biotechnology, Gdansk, Poland) and transferred to HPLC vials for analysis. The analysis of photosynthetic pigments was performed using an Agilent 1260 Infinity Series HPLC system (Agilent Technologies, Inc., Santa Clara, CA, USA) equipped with a refrigerated autosampler, thermostatted column compartment, quaternary pump with an in-line vacuum degasser, and a photo-diode array detector (PDA). Pigment separation was carried out on an Eclipse XDB C8 column (150 × 4.6 mm; Agilent Technologies, Inc., Santa Clara, CA, USA) maintained at 25 °C. Visible absorption spectra were recorded within the range of 350–750 nm. The injection volume was 200 μL. Mobile phase A consisted of methanol, acetonitrile, and 0.25 M aqueous pyridine solution (pH 5.0) in a ratio of 50:25:25 (*v*/*v*/*v*), whereas mobile phase B contained methanol, acetonitrile, and acetone in a ratio of 20:60:20 (*v*/*v*/*v*). Pigment separation was achieved using a linear gradient from 100% mobile phase A to the initial conditions at the 40th minute of the analysis, according to Zapata et al. [71]. The flow rate was maintained at 1 mL min^−1^. Chromatographic data were processed using ChemStation C.01.09 software (Agilent Technologies, Inc., Santa Clara, CA, USA).

### 4.7. Determination of Oxidative Stress Markers

Hydrogen peroxide (H_2_O_2_) content in *C. vulgaris* and *S. quadricauda* cells was determined spectrophotometrically according to the method described by Alexieva et al. [72]. Briefly, 10 mL of algal suspension was centrifuged, and the resulting pellet was homogenized in 0.1% (*w*/*v*) trichloroacetic acid (TCA). After centrifugation, the supernatant was mixed with potassium phosphate buffer (pH 7.0) and potassium iodide (KI). The absorbance of the reaction mixture was measured at 390 nm. Hydrogen peroxide concentration was calculated using a calibration curve prepared with known H_2_O_2_ concentrations and expressed on a per-cell basis. Lipid peroxidation was estimated by measuring malondialdehyde (MDA) content using the method of Heath and Packer [73]. Briefly, 10 mL of algal suspension was centrifuged, and the pellet was homogenized in 0.1% (*w*/*v*) TCA. Following centrifugation, the supernatant was mixed with thiobarbituric acid (TBA) prepared in trichloroacetic acid and heated at 95 °C for 30 min. The reaction was terminated by rapid cooling in an ice bath, and the samples were centrifuged to remove insoluble material. Absorbance was measured at 532 and 600 nm, and MDA concentration was calculated using an extinction coefficient of 155 mM^−1^ cm^−1^. The results were expressed on a per-cell basis.

### 4.8. Determination of Non-Enzymatic Antioxidants

The ascorbate and proline contents as indicators of non-enzymatic antioxidants were determined spectrophotometrically. Ascorbate content was determined according to the method of Kampfenkel et al. [74]. Briefly, 10 mL of thoroughly mixed algal suspension was centrifuged (2000 rpm, 10 min), and the resulting pellet was extracted with 5% metaphosphoric acid. After centrifugation (4000 rpm, 10 min), 0.2 mL of the supernatant was mixed with 0.2 mL of either ascorbic acid standard solution or distilled water; then, 1.0 mL of Folin–Ciocalteu reagent diluted 1:10 with distilled water and 0.2 mL of 1 M NaOH were added. The reaction mixtures were incubated for 10 min, and absorbance was measured at 750 nm. Ascorbate concentration was determined using a calibration curve prepared with ascorbic acid standards and expressed per cell. Proline content was determined according to Bates et al. [75]. Algal pellets obtained after centrifugation were homogenized in 5 mL of 100 mM potassium phosphate buffer, pH 7.5 and centrifuged (3500 rpm, 10 min). Subsequently, 0.5 mL of the supernatant was mixed with 0.5 mL of 1% ninhydrin solution prepared in 60% acetic acid and 0.5 mL of proline standard solution or buffer. The reaction mixtures were incubated at 100 °C for 15 min, cooled to room temperature, and extracted with 2 mL of toluene. After vigorous mixing, the absorbance of the upper organic phase was measured at 520 nm. Proline concentration was calculated using a calibration curve prepared with proline standards and expressed on a per-cell basis.

### 4.9. Determination of Antioxidant Enzyme Activities

Crude enzyme extracts were prepared from algal cells of *C. vulgaris* and *S. quadricauda* according to previously described procedures. Briefly, 10 mL of thoroughly mixed algal suspension was centrifuged (2000 rpm, 10 min, 4 °C), and the supernatant was discarded. The resulting pellet was homogenized in an ice-cold extraction buffer containing 0.05 M potassium phosphate buffer (pH 7.0), 0.1 mM EDTA, and 1% (*w*/*v*) polyvinylpyrrolidone (PVP). Homogenates were centrifuged (2000 rpm, 10 min, 4 °C), and the resulting supernatants were used for enzyme activity assays. Ascorbate peroxidase (APX) (EC 1.11.1.1) activity was determined according to Nakano and Asada [76] by monitoring the decrease in absorbance associated with ascorbate oxidation at 290 nm. The reaction mixture contained potassium phosphate buffer (pH 7.0), sodium ascorbate, hydrogen peroxide, and enzyme extract. Enzyme activity was calculated using the extinction coefficient of 2.8 mM^−1^ cm^−1^. Catalase (CAT) (EC 1.11.1.6) activity was determined following Aebi [77], measuring the decomposition of hydrogen peroxide at 240 nm. The reaction mixture consisted of potassium phosphate buffer (pH 7.0), H_2_O_2_, and enzyme extract. Catalase activity was calculated using the extinction coefficient of 45.2 mM^−1^ cm^−1^. Superoxide dismutase (SOD) (EC 1.15.1.1) activity was assayed according to [78] based on the inhibition of nitroblue tetrazolium (NBT) photoreduction. The reaction mixture contained potassium phosphate buffer (pH 7.0), methionine, riboflavin, EDTA, NBT, and enzyme extract. The reaction was initiated by exposure to light and terminated by transferring the samples to darkness. Absorbance was measured at 560 nm. One unit of SOD activity was defined as the amount of enzyme required to inhibit NBT photoreduction by 50%. Glutathione reductase (GR) (EC 1.8.1.7) activity was determined according to Schaedle and Bassham [79] by monitoring the oxidation of NADPH at 340 nm. The reaction mixture consisted of potassium phosphate buffer (pH 7.0), oxidized glutathione (GSSG), EDTA, NADPH, and enzyme extract. Enzyme activity was expressed as the rate of NADPH oxidation.

### 4.10. Physicochemical Characterization of HDPE Microplastics Following Algal Exposure

Changes in the physicochemical properties of HDPE microplastics following exposure to *C. vulgaris* and *S. quadricauda* were evaluated using complementary analytical techniques. Surface chemical modifications were assessed by Fourier-transform infrared spectroscopy (FTIR) spectroscopy [34]. Morphological changes in HDPE particles were examined using scanning electron microscopy (SEM). In addition, porosimetry analyses were performed to determine the specific surface area and pore characteristics of HDPE particles before and after algal exposure. FTIR measurements were performed using a Nicolet 6700 spectrometer (Thermo Scientific, USA) operating in ATR mode. To register FTIR spectra, a small amount of dried sample was placed on the diamond surface and pressed against the ATR crystal using a pressure clamp. Surface morphology analysis was carried out using an Inspect S50 scanning electron microscope (FEI, USA) [34]. Prior to SEM analysis, microplastic samples were mounted on aluminum SEM stubs using conductive carbon tape. Due to the insulating nature of polymeric materials, the samples were sputter-coated with a 5 nm gold layer using a Leica EM sputter coater to improve electrical conductivity and minimize charging effects during imaging. Nitrogen adsorption–desorption measurements were performed using an ASAP 2020 surface area and porosity analyzer (Micromeritics, USA). Prior to analysis, approximately 0.2 g of each sample was degassed under vacuum conditions (2 mmHg) at 100 °C for 15 h to remove adsorbed moisture and volatile impurities. The textural properties were determined from nitrogen adsorption–desorption isotherms measured at −196 °C. The specific surface area was calculated using the Brunauer–Emmett–Teller (BET) method, while the pore size distribution was evaluated using the Barrett–Joyner–Halenda (BJH) method. Analyses were performed on virgin HDPE particles and on MPs recovered after 5 days of exposure to the respective microalgal cultures [34].

### 4.11. Statistical Analysis

Each measurement was performed in four replicates, and each experiment was repeated independently at least twice. Prior to statistical analyses, the data were tested for normality using the Shapiro–Wilk test and for homogeneity of variances using Levene’s test. No significant outliers were detected in the datasets. Since the assumptions of normal distribution and homogeneity of variances were met, differences among treatments were analyzed using one-way analysis of variance (ANOVA). When significant effects were detected, Tukey’s post hoc test was applied to compare treatment means. All statistical analyses were performed using IBM SPSS Statistics 21 (IBM Corp., Armonk, NY, USA). Differences were considered statistically significant at *p* < 0.05.

## 5. Conclusions

Our results provide important insights into the effects of both particle sizes, 40 µm and 125 µm of HDPE on *C. vulgaris* and *S. quadricauda* growth, biochemical component levels, oxidative stress, and antioxidant response and improve the present knowledge of the potential toxicity of MPs in studied freshwater organisms. HDPE treatment negatively correlates with cell number in both algal cultures and the levels of organic compounds such as chlorophylls, monosaccharides and proteins. In contrast, the activity of antioxidant enzymes and the accumulation of non-enzymatic antioxidants (carotenoids, ascorbate, and proline), which are directly or indirectly involved in ROS scavenging, were significantly increased in algal cells treated with both HDPE particle sizes. An amount of 40 µm HDPE was more effective in activating the antioxidant system in comparison with a larger particle size of HDPE (125 µm), whereas 125 µm HDPE was characterized by a higher negative effect on algal growth and metabolism in relation to a smaller HDPE particle size (40 µm), indicating a different toxic mechanism of these two particles of MP. The results show that both *C. vulgaris* and *S. quadricauda* induced physicochemical modifications of the HDPE surface. Our findings highlight the importance of algal–microparticle interactions in determining cellular and environmental responses, confirm MP phytotoxicity, inform future risk assessments under realistic aquatic conditions, and contribute to effective methods of plastic waste management in water treatments. Understanding the defense mechanisms of microalgae against HDPE can help elucidate the ecotoxic effects of micropollutants and the bioremediation potential of green microalgae.

## Figures and Tables

**Table 1:** The effect of HDPE (40 and 125 µm) at the highest concentration of 10 mg L^−1^ on carotene and xanthophyll contents in *Chlorella vulgaris* and *Scenedesmus quadricauda* cells on the 5th day of cultivation in relation to the control. Results show the mean values of four independent determinations with the respective standard deviations. Treatment with at least one letter the same is not significantly different according to Tukey’s post hoc test.

Pigment (fg Cell^−1^)	*Chlorella vulgaris*		
	Control	40 µm HDPE	125 µm HDPE
Astaxanthin	2.733 ± 0.155 ^b^	3.267 ± 0.035 ^a^	2.551 ± 0.014 ^c^
*α*-Carotene	4.734 ± 0.014 ^b^	5.109 ± 0.016 ^a^	4.016 ± 0.073 ^c^
*β*-Carotene	6.186 ± 0.051 ^b^	6.918 ± 0.161 ^a^	6.105 ± 0.103 ^b^
Cryptoxanthin	5.481 ± 0.105 ^b^	6.725 ± 0.107 ^a^	4.309 ± 0.055 ^c^
Lutein	2.545 ± 0.044 ^b^	2.989 ± 0.097 ^a^	1.808 ± 0.026 ^c^
Neoxanthin	8.483 ± 0.021 ^b^	9.262 ± 0.036 ^a^	7.051 ± 0.017 ^c^
Violaxanthin	3.067 ± 0.042 ^b^	4.122 ± 0.018 ^a^	2.598 ± 0.013 ^c^
Zeaxanthin	11.377 ± 0.025 ^b^	14.443 ± 0.335 ^a^	10.226 ± 0.218 ^c^
Pigment (fg Cell^−1^)	*Scenedesmus quadricauda*		
	Control	40 µm HDPE	125 µm HDPE
Astaxanthin	2.437 ± 0.083 ^b^	3.889 ± 0.142 ^a^	2.001 ± 0.015 ^c^
*α*-Carotene	4.611 ± 0.015 ^b^	5.504 ± 0.105 ^a^	3.805 ± 0.039 ^c^
*β*-Carotene	6.002 ± 0.028 ^b^	7.917 ± 0.098 ^a^	5.116 ± 0.009 ^c^
Cryptoxanthin	5.024 ± 0.018 ^b^	6.295 ± 0.066 ^a^	4.406 ± 0.023 ^c^
Lutein	2.388 ± 0.107 ^b^	3.603 ± 0.116 ^a^	1.258 ± 0.076 ^c^
Neoxanthin	8.115 ± 0.026 ^b^	9.774 ± 0.702 ^a^	6.995 ± 0.028 ^c^
Violaxanthin	2.784 ± 0.043 ^b^	3.155 ± 0.082 ^a^	2.001 ± 0.052 ^c^
Zeaxanthin	11.004 ± 0.058 ^b^	14.691 ± 0.117 ^a^	9.511 ± 0.061 ^c^

**Table 2:** Summary of porosimetry analysis and BET specific surface area of virgin HDPE and HDPE after cultivation with algae on the 5th day of the experiment. The results show the mean values of four independent determinations, along with their respective standard deviations. Treatment with at least one letter the same is not significantly different according to Tukey’s post hoc test.

HDPE Variants	BET [m^2^ g^−1^]	Maximum Pore Width [nm]
HDPE 40 µm	0.31 ± 0.008 ^d^	68 ± 3.251 ^f^
HDPE 40 µm + *Chlorella vulgaris*	5.97 ± 0.043 ^a^	191 ± 0.862 ^b^
HDPE 40 µm + *Scenedesmus quadricauda*	3.50 ± 0.034 ^c^	185 ± 1.347 ^c^
HDPE 125 µm	3.54 ± 0.026 ^c^	71 ± 0.609 ^e^
HDPE 125 µm + *Chlorella vulgaris*	3.50 ± 0.057 ^c^	197 ± 0.905 ^a^
HPE125 125 µm + *Scenedesmus quadricauda*	4.07 ± 0.09 ^b^	135 ± 2.068 ^d^

## Data Availability

Data are contained within the current article.

## Abbreviations

The following abbreviations are used in this manuscript:

APX	Ascorbate Peroxidase
BET	Brunauer–Emmett–Teller
CAT	Catalase
FTIR	Fourier-Transform Infrared Spectroscopy
GR	Glutathione Reductase
HDPE	High-Density Polyethylene
MDA	Malondialdehyde
MP	Microplastic
PA	Polyamide
PE	Polyethylene
PET	Polyethylene Terephthalate
PP	Polypropylene
PS	Polysterene
PVC	Polyvinyl Chloride
ROS	Rective Oxygen Species
SEM-EDX	Scanning Electron Microscopy Coupled with Energy-Dispersive X-Ray Spectroscopy
SOD	Superoxide Dismutase
